# A Qualitative Mixed-Method Narrative Study on Psychotherapeutic Support Needs Based on a Series of 11 Cases of Survivors of the 2023 Odisha Train Accident

**DOI:** 10.7759/cureus.81161

**Published:** 2025-03-25

**Authors:** Sarada P Swain, Sushree S Behura, Shraddha Banerjee, Sukanya Mahakul, Nilamadhab Kar

**Affiliations:** 1 Psychiatry, Srirama Chandra Bhanja Medical College, Cuttack, IND; 2 Clinical Psychology, Srirama Chandra Bhanja Medical College, Cuttack, IND; 3 Clinical Psychology, SCB Medical College, Cuttack, IND; 4 Special Education Need Globe, Kalinga Institute of Industrial Technology (KIIT) International School, Bhubaneswar, IND; 5 Research Institute in Health Sciences, University of Wolverhampton, Wolverhampton, GBR; 6 Faculty of Contemplative and Behavioural Sciences, Sri Sri University, Cuttack, IND; 7 Psychiatry, Black Country Healthcare NHS Foundation Trust, Wolverhampton, GBR

**Keywords:** health services, mental health services, passenger, posttraumatic stress, psychological stress, psychotherapy, railroad, survivor, train accident

## Abstract

Background: Rail and road accidents are common in India and are extremely stressful life events. Many accident survivors develop stress-related mental health problems but do not get psychotherapeutic support. We intended to analyse the needs and types of techniques for the psychotherapeutic support of the survivors of the 2023 train accident in Odisha and to reflect on how the services can be facilitated.

Methods: It was a qualitative, mixed-method, narrative study based on the interview of a sample of 11 survivors of the train accident.

Results: It was observed that the survivors and their families had many unresolved psychological issues related to the trauma of the train accident and its consequences. The survivors articulated their mental health concerns holistically in a comprehensive way. It appeared that their psychotherapeutic needs were unmet. Examples of psychological interventions needed were psychoeducation, relaxation, supportive and cognitive therapies, and specific trauma-focused cognitive behavioural therapy. Many challenges in providing psychotherapies were identified, such as limited awareness about the need for psychotherapeutic intervention, affected persons in geographically highly dispersed areas, unavailability of psychotherapeutic services or personnel in most places, and lack of resources.

Conclusion: The train accident survivors have immense psychotherapeutic needs, but these are mostly unmet. Modifying the provision methods to tele-psychotherapy and training other healthcare personnel such as nurses, and counsellors might help the resource-scarce situation.

## Introduction

Train accidents are common in India [1]. On 2nd June 2023, there was a major train accident in the Balasore district of Odisha, India. It was a three-train collision, where reportedly 296 people died and more than 1200 were injured [2]. There was a massive rescue effort by the authorities and the injured survivors were taken to nearby medical facilities for medical interventions [3]. Financial support was given by the governments to injured passengers and the families of the deceased passengers. Beyond this, there was no other specific support available for the affected individuals.

Not only the passengers but also the rescuers and family members had a traumatic experience through this train accident. It is well known that such catastrophic traumatic events lead to mental health problems such as anxiety, depression, and posttraumatic stress among a proportion of survivors. Specifically, there are reports of stress-related mental illnesses such as adjustment disorders, posttraumatic stress disorders (PTSD), anxiety and depression disorders following train accidents [4]. In addition, some of the survivors of railroad and traffic accidents have life-changing physical and cognitive disabilities secondary to fractures, physical injuries, and traumatic brain injury. There are various secondary stressors as well, such as disability-related job loss, financial problems, etc. These consequences often lead to long-term psychological problems.

Post-trauma psychotherapies

Considering the frequency of train accidents, the information about the mental health consequences of the survivors and other indirectly affected people is limited. The reported psychiatric diagnoses include mostly PTSD [5-7] although anxiety and depressive disorder are also common. There is hardly any study on the psychotherapeutic need and intervention of train crash survivors; although psychotherapy for train drivers following railway suicides has been reported [8]. Experience from an acute mental health crisis response following a train accident reported that only a few required counselling; exploration and reassurance were helpful and adequate; a helpline was set up but was not used [9]. They also reported staff debriefing and suggested developing a compact responsive team.

It cannot be overemphasized that there is a need for psychological assessment and support for train accident survivors. It has been suggested that survivors who were injured, experienced risk of death, felt trapped, or witnessed death would require long-term psychological input [10]. There are many therapies specific for trauma survivors, especially those who develop stress-related psychiatric disorders such as PTSD. These psychotherapies include cognitive therapies, trauma-focused cognitive behavioural therapy (CBT), etc. [11]. All these can also be useful for the affected survivors of the train accidents.

Rationale of the study

Despite a huge number of rail and traffic accidents, there are hardly any studies evaluating the psychological support needs of these survivors. There is no study about psychotherapeutic methods appropriate for this group of individuals. There are challenges in assessing and providing psychological interventions as the passengers belong to highly dispersed geographical areas. However, there are facilities for psychological interventions such as in the psychiatry departments of medical colleges and private setups.

Aim

In this context, we intended to evaluate the clinical presentations of the survivors of the 2023 Odisha train accident to ascertain the needs and types of techniques for psychological intervention that would be required. It was expected that this would help to reflect on possible ways of providing such psychotherapy services for the survivors in a resource-scant region with limited availability of mental health services.

## Materials and methods

The study was conducted in the Department of Psychiatry, Mental Health Institute, Cuttack around three months after the 2023 Odisha train accident. It was part of the project to provide psychosocial support for the accident survivors. The assessments were carried out over the phone, which started in September 2023.

The participants included the passengers who were medically assessed or treated at the Sriram Chandra Bhanj Medical College Hospital, Cuttack for accident-related injuries. They were contacted serially from the list of 164 survivors available at the hospital; in this process, 58 passengers could be assessed, and 11 of them agreed to participate and engage in this study.

It was a qualitative, mixed-method, narrative study based on the interviews of the survivors of the train accident. The psychological assessments were carried out by three clinical psychologists who had Master in Philosophy degrees in clinical psychology; and were working in the Department of Psychiatry. The clinical psychologists assessed the survivors over the phone, in the first language of the survivors, which varied between Odia, Hindi, and Bangla. The sessions were 45-60 minutes long usually, however some continued longer allowing for the emotionally distressed state of the survivor or additional information from the family caregivers. The psychological assessments continued for up to two sessions.

The assessments had a temporal approach, with cross-sectional and longitudinal components. In the cross-sectional component, the reviews captured the emotional state and experiences of the survivors at the specific moment of the accident; and the longitudinal component involved the studying evolutions of psychological symptoms over a period following the accident and during the sessions that allowed for formulating the individual emotional needs, at the end of which a psychotherapeutic intervention plan was suggested. When available and consented by the survivors, their family caregivers were interviewed as well. The family caregivers provided the collateral history of the mental health status of the survivors. This improved the understanding of the current mental state of the passenger from a different perspective. In addition, many family caregivers were also psychologically stressed and found this interaction helpful.

There were options for attending psychotherapy sessions in person at the department or over the phone. However, all the reviews happened through the phone. These were one-to-one individual assessments. The results of the psychological assessment and intervention plan are presented as a case series.

The review process involved providing information about the project; explaining the reasons for assessment and the psychosocial support available. The reviews were facilitated by some key questions, such as the individual experience of the train accident, initial psychological reaction, later psychological symptoms related to this, impact on functioning, relationships, and any other areas of life. It was also enquired about any support received and, the availability and accessibility of any local psychotherapeutic resources.

The transcript of the interview content was analysed for the themes, which included psychological reactions and concerns, additional stress, and needs. This process was supervised and the contents were discussed among the psychology and research teams. Following these, the psychotherapeutic techniques were reflected and agreed upon through a consensus method.

The study was approved by the Institutional Ethics Committee (No: 1456; Date 16/08/2023). During the review, it was highlighted that psychosocial intervention would be provided irrespective of the participation in the study. Anonymity and the option to withdraw at any time without assigning any reason were highlighted. Those who agreed to participate in the study following the discussion were requested to provide informed consent verbally and through an electronic format.

## Results

Trauma narratives by the participants

The train accident as experienced by the passengers was described in various ways. It was narrated as suddenly everything turned dark, and there was an enormous noise. People fell over others; some were unconscious or possibly died instantly. Passengers, who could manage to come out, realized the severity of the accident, that coaches were stacked on top of one another; there were twisted steel and severed limbs strewn across the torn-up metal debris; and passenger goods were scattered everywhere.

People were helping to pull other passengers out, some were crying, frantically searching for loved ones. Sometime later emergency personnel arrived and were moving people out to safety, and gathering the dead bodies in rows, while more passengers were being pulled from the wreckages. There were many unconscious people, who were probably considered dead and left among dead bodies. There was a horrific spectacle of relatives lugging the mutilated bodies of their departed loved ones; ‘limbs strewn everywhere - a hand here, a leg there - someone’s face was deformed.’

Initial psychological reactions

‘When the tragedy struck, we thought we were dead. As soon as we realized we were still alive, we started to move toward the emergency window, so we could get off the train.’ Many described the experience as nightmarish and there was pandemonium everywhere. A few were speechless and dumbfounded. A few passengers and family members helped others, showing signs of optimism and expectation that they might still be alive. Many had an immediate relief of having survived such a horrific crash when many perished. Some people were thankful to God. Some described that even if they lost personal items, important papers, or documents, they were thankful that they were still alive. An immediate worry for those injured was how the injuries would be treated, and whether they could recover completely.

Many passengers got separated from their families who were travelling together, and other co-passengers; and there was intense distress of not knowing what had happened to them. A mother described while lying in a hospital bed, she begged the staff to locate her brother and son, who she later learned had passed away. Distress of being separated from the family members who were travelling together, taken to hospital, not knowing their fate for days on end was most stressful in the context of so many people were dead; and were being treated in various hospitals far apart. Most wanted nothing more than to be reunited with their family at that time. For variable lengths of time, many passengers could not contact their families, and this was also reported as painful.

Physical injuries and impact

The impact of the crash in the form of injuries was hugely evident. There was severe bleeding, multiple injuries, single and multiple fractures in various places, loss of consciousness, and acute pain. The injured passengers were taken to nearby hospitals and some with more serious injuries were shifted to hospitals far away, considering the lack of facilities close by. Many people underwent surgical and orthopaedic operations for injuries and some of them required internal fixation for fractures. The interventions required the survivors to stay in hospitals or intensive care units for several days and weeks. While some were happy and full of gratitude regarding the treatment they received in the hospital, some were not. Many injured passengers continued to have persistent pain, mobility problems, and disabilities, which affected their capacity to join back their usual work. This led to financial dependence on others and worries. Some passengers were thankful to the government and other agencies, for financial and other assistance.

Stress symptoms

Some were unable to remember what had happened, and how they were rescued and supported. It could be due to impaired sensorium, losing consciousness, or amnesia secondary to severe psychological trauma. The accident memories were very traumatic when they were described. Passengers said they were haunted by dreams, and there were sleep disturbances. When they recalled, they had palpitations and became very anxious. Most were feeling low and disinterested in their usual life. Many reported irritability, anger, agitation, and isolating themselves leading to decreased socialization. Some reported having decreased interest in physical relationships. A few passengers felt that there was a lack of understanding from their family, and spouse, about their changed state and felt humiliated by their remarks. A few continued to worry about the disabilities and whether they could recover to work appropriately to be financially independent.

Case series

Case 1

Presentation: A male, 33, presented with anxious preoccupations, fearfulness, and apprehensions. He described, ‘My heartbeat increases if I hear any news of accidents by any means’, and ‘I am very fearful of travelling after that incident’. He shared, ‘I am very worried about my future and how to sustain my family?’ He had an operation for a fractured leg and was still not able to walk properly. He mentioned, ‘I get irritable with small matters’ and that leads to anger outbursts (at times). There was decreased social interaction, ‘I do not feel like talking to anyone and like to stay alone nowadays’.

Psychotherapeutic considerations: The anxious state suggested the consideration of relaxation therapy. As the relaxation technique diaphragmatic breathing exercise was chosen following the discussion. These might help to deal with anxiety and apprehension. The anxious preoccupations and distorted thoughts required CBT. In addition, the management of anger outbursts was required and was based on behavioural techniques, such as distraction, time out, etc. All these might help improve his social interaction, however, if there were still concerns regarding his socialization further evaluation and if needed, social skill training might be appropriate.

Case 2

Presentation: A male, 31, reported persistent low mood, irritability, and forgetfulness. He reported that dreams related to the accident disturbed his sleep cycle and he felt helpless. He had negative thoughts related to self, such as; ‘I am incapable of working and earning money’, ‘I am dependent on others for favour’ etc. He was also distressed by the physical pain due to the injuries he had during the accident. His appetite was decreased.

Psychotherapeutic considerations: Considering the high level of distress, supportive therapy, appropriate space for venting, and relaxation therapies were considered at the initial stage followed by CBT which was planned to address the negative cognitions related to low self-esteem and his thoughts of helplessness. Further evaluation of sleep and nightmares suggested sleep hygiene-related counselling and CBT for insomnia. He was also suggested to engage in feasible activities such as light walking, work that he can do from home, and socialization, considering his physical health condition.

Case 3

Presentation: A male, 28, was injured during the train accident. He shared about his apprehensions and worries related to the future. He was facing movement difficulty which became an obstacle for him to earn his living. He felt incapable. He shared, ‘I feel my life has been ruined. It can’t be better again. I have to live with these physical pains. I do not feel any happiness inside’. ‘All the time, I think of my condition and feel pity’. He added, ‘I do not feel like doing anything or talking to anyone’.

Psychotherapeutic considerations: Cases 3 and 4 were a married couple. The plan for psychotherapeutic intervention involved both of them and included relaxation exercises, to manage anxiety and restlessness and CBT to address the negative thought processes found in both husband and wife. Specific therapies for him included problem-solving strategies as he shared issues and worries about his work-related problems. This would help him arrive at practical solutions feasible in the current situation he was in. He would need employment support for his work considering the pain and mobility problems. Emergent issues following further assessment suggested social skills training for dealing with issues at the workplace and interpersonal psychotherapy.

Case 4

Presentation: A female, 29 was injured during the train accident. She said that whenever images related to the day of the accident came to her mind, she would start feeling restless and anxious and would have palpitations. Thoughts such as, ‘Things can’t be better again’ and ‘our life is completely disturbed after the accident’ recurred. She reported that after this incident her conjugal life had become very disturbed.

Psychotherapeutic considerations: The suggested psychotherapy plan was relaxation therapy and CBT initially to deal with anxiety states and cognitive distortions. After these, couple therapy was suggested to address marital problems. There were communication issues as well related to accident trauma, mobility; and work-related issues of the husband which were additional stressors for the person.

Case 5

Presentation: A male, 31, presented with feeling low most of the time of the day, decreased interest in socializing, and feelings of irritability and agitation due to persistent physical pain secondary to the injury during the train accident. He had undergone surgery. He had recurrent thoughts, such as; ‘I am the victim of destiny’, ‘How could such an incident happen to me’, etc. He had an intense traumatic experience in the accident, as he was mistakenly placed with the dead bodies by the rescue personnel, as he was in an unconscious state. Later when he regained consciousness, he was shaken by seeing dead bodies around him. His psychological distress was aggravated further by occasional critical comments, such as, ‘You are of no use now’, and humiliation from his wife due to his unemployment and financial constraint; which disturbed their marital relationship.

Psychotherapeutic considerations: Supportive therapy and facilitation of venting were the focus during the initial phase of the intervention. Subsequently, CBT was planned to address his cognitive distortions which were evident. His physical pain was an important contributor to his psychological distress. Problem-solving with brainstorming was considered, for the support about physical issues. He was encouraged to socialize; social skill training for his inhibition with changed circumstances was planned. Psychoeducation of his wife was conducted for her expressed emotions and their impact.

Case 6

Presentation: A male, 32, had anxious preoccupations, feeling very low, with negative thinking about himself. He mentioned, ‘I do not feel from inside to talk to anyone or to do some work’; ‘I like to stay silent in my own space’. He was under persistent physical pain due to the injury during an accident which was making him more irritable and angry.

Psychotherapeutic considerations: During the assessment, it was observed that he was an introverted person and was not expressive by nature. The psychotherapeutic plan included supportive therapy for his emotional distress, CBT for depressive cognitions, activity scheduling; and problem-solving strategies were also included.

Case 7

Presentation: A male, 29, presented with anxiety, fear, and trauma-related symptoms due to the experiences he had in the train accident, which were recurrent. He was thankful to God that he survived and tried helping other passengers in the immediate aftermath of the accident. His memory of witnessing relatives hugging mutilated body parts left him utterly shocked and speechless. He said, ‘Flashes from that day keep coming to my mind. It’s horrifying’. He said that he remained worried all the time about his future. Dysfunctional thoughts could be identified in his thought process.

Psychotherapeutic considerations: The psychotherapy plan included the facilitation of venting as a supportive therapy, where he talked about the traumatic experience. Relaxation exercises were emphasized in the context of anxiety and fear. Trauma-focused psychotherapy was conducted where cognitive, behavioural, and emotional aspects related to the event were addressed. Principles of CBT were extensively used in the process.

Case 8

Presentation: A female, 30, was travelling with her child on that day. During the accident, with sudden jerks, she got separated from her child. After gaining consciousness, she was extremely apprehensive and had overwhelming emotions as she did not find her child. She lost her child in the train accident. During the evaluation, she had persistent low mood, frequent crying spells, suicidal ideas, and cognitions such as ‘There is no point of living without my child, I don’t want to live’.

Psychotherapeutic considerations: Supportive therapy, facilitating her to vent was the initial step. The further psychotherapeutic process included trauma-focused-psychotherapy with cognitive and behavioural components along with support for the emotional aspects that were addressed from time to time. Overwhelming emotions were approached through dialectical behavioural therapy (DBT) techniques.

Case 9

Presentation: A male, 31, presented with worrisome, anxious preoccupation, apprehension, and restlessness due to disturbed thoughts. Cognitions such as, ‘travelling by any means is not safe’, and ‘everything has ruined after this incident, now nothing will be like before’ were shared. He reported persistent low mood and a feeling that he can’t be happy again. He had decreased social interactions, ‘I don’t want to face anyone because they will ask about my present state’.

Psychotherapeutic considerations: Initial psychotherapeutic sessions were supportive therapy. The assessment revealed many distortions in thought processes leading to low mood. CBT, activity scheduling, and engaging him in productive activities were considered. His inhibition to interact with others was dealt with through cognitive therapy and social skill training.

Case 10

Presentation: The male, 35, was not able to overcome the traumatic experiences during the train accident. Flashbacks were disturbing. He had anxious preoccupations; he had dreadful cognitions related to medical conditions. Some examples of his cognitive distortions were: ‘I am not able to earn a living for my family’ or ‘I don’t know if I will be able to be functional again like before’. There were decreased social interactions due to his physical limitations after the injury. He was hesitant to face people because he felt he was no longer a ‘capable man’.

Psychotherapeutic considerations: In the context of such intense trauma, supportive therapy, trauma-focused psychotherapy, and activity scheduling were considered. Cognitive restructuring techniques are used as part of CBT to address distorted cognitions. Overwhelming emotions were handled through DBT techniques. Socialization was encouraged.

Case 11

Presentation: A male, 28, had extremely traumatic experiences during the train accident. He thought he was dead when the accident happened. He felt fortunate that amidst so many dead he was alive. He had anxious preoccupations, apprehensions, and fearful dreams about the accident. He was restless, agitated, and had palpitations for some time. He shared that he was scared of travelling by train or even by car due to his experience. He felt anxious or palpitations with trivial matters and this was hampering his work. He remained over-cautious while at work or in a social setting, protecting himself from getting hurt by anything physically.

Psychotherapeutic considerations: Psychotherapeutic interventions were modelled around his anxiety and traumatic symptoms. Supportive therapy sessions were conducted in the context of traumatic experiences, along with relaxation techniques, primarily through breathing exercises based on yoga. CBT was used to address his negative cognitions leading to anxious feelings and cognitions.

## Discussion

The study tried to put together the trauma experienced by the passengers of the 2023 train accident in India, described the psychological impact, and reflected on the psychotherapeutic need. Case reports of 11 survivors formed the information base of this qualitative study.

The trauma

The degree of trauma was catastrophic; it was sudden without any pre-warning or chance to protect self. While some described a relief that they survived this accident, the physical and mental consequences were extreme. There were classic stress symptoms, along with anxiety and depressive symptoms, suggestive of stress-related psychiatric illnesses.

Most of the passengers travelled with family members or in groups. There was an added stress of missing them during the chaos immediately after the accident as it was dark, and there was a huge commotion. There was difficulty in communicating, as passengers were triaged to different hospitals, and it was not possible to get information about the whereabouts of the passengers, especially the unidentified ones. This stressor of not knowing where the family members were and their outcome, with the possibility of death, was an extremely difficult situation for many passengers and their family members. Additionally, finding oneself among dead bodies, misidentification of bodies, and challenges of non-identification were also present.

The psychological impact

In the immediate aftermath, mutilated dead bodies of family members and other passengers created an extremely gory scenario. There was a sense of intense fear, and helplessness, especially for the injured passengers before being rescued. There was concern about whether they would survive or not. Later survivors did receive the required medical treatment and surgeries, but reportedly complications continued and disabilities related to mobility and pain persisted. Passengers who got admitted, stayed in the hospital for days and weeks, before they could be discharged.

Months after the accident when the survivors were contacted for the psychological assessment, it was observed that the memories of that incident were still strong affecting survivors and their families in multiple spheres. Traumatic memories of the accident were being relieved by many survivors, and they were distressed with many posttraumatic symptoms, such as anxiety, depression, irritability, anger, phobia, insomnia, nightmares, helplessness, diminished self-confidence, etc. There were considerable functional impairments; many were unemployed and unable to perform other usual daily activities.

Until the survivors talked to the clinical psychologists, they had no scope to share their mental state. On the contrary many of their psychological presentations were not understood by the family or spouse. The interaction provided an opportunity for the survivors to vent, to be understood, and to be validated that these reactions were the usual consequences of such catastrophic accidents.

Distress of family caregivers

Alongside the survivors, their family members had immense psychological stress. Not knowing the state of their loved ones, not being able to find them in the hospital in the initial days, fear of the worst outcomes, and non-identification to misidentification of dead bodies, were many such contributing factors. In the hospitals, the concerns were about the outcome of the treatment for multiple injuries, the risk of long-term disability, etc. Later, they had to deal with the issues of psychological symptoms, such as irritability, frequent mood swings, anger outbursts, persisting anxiety, depression, etc. Additionally, there was isolation, joblessness, financial struggles, inadequate support from the authorities, etc. Many family caregivers reported their own mental health symptoms as well.

Psychotherapy assessment outcomes

The interactive sessions with survivors and their family members provided a lot of information about the trauma, and the psychological state of the survivors and their families, and the experience was overwhelming for many. The survivors had piled up emotions and were finding it difficult to make others understand. Even the family members appeared exhausted from listening to their grievances. During therapeutic assessments when they got a chance of venting, it appeared as if they spoke their heart out. During the initial phase of interaction with the clinical psychologists, they expressed their fear related to the accident, their worries, feelings of helplessness, and worthlessness. They were also distressed not only about the injuries which were taking time to heal, but also their overall state.

It was clear that there was a need for psychological interventions and considering the complex situations, different types of therapies and approaches were required. A summary of therapies considered is given in Table 1. It is expected that most of these would be relevant for the affected persons including survivors and family caregivers. There was clearly a need for psychoeducation about trauma and its associated symptoms, supportive psychotherapeutic strategies, CBT, and specifically trauma-focused CBT, etc. It became evident that for various associated issues, secondary stressors, and their impact; other therapeutic approaches would be required. The psychotherapeutic sessions are expected to be multiple, continuing for a considerable period. 

**Table 1:** Summary of psychotherapeutic strategies considered

Type of psychological strategies
Activity scheduling
Anger management
Behavioural activation
Cognitive behavioural therapy
Cognitive behavioural therapy for insomnia
Couples therapy
Dialectical behaviour therapy
Family support
Interpersonal psychotherapy
Problem-solving therapy
Psychoeducation
Relaxation therapy
Sleep hygiene counselling
Social skill training
Supportive therapy
Trauma-focused cognitive behavioural therapy
Trauma-focused-psychotherapy

Psychotherapeutic interventions were also needed for the family members, who were stressed and indirectly affected by the incident. Many of them were struggling which was having an impact on their interpersonal relationship and understanding. A psychotherapeutic treatment plan would include stress management education, and other specific therapies as appropriate dependent on their psychological presentation.

Challenges for psychotherapeutic services

The assessments highlighted the enormous need for psychological intervention for both the directly and indirectly affected persons in the train accidents. However, many challenges remain, which are related to limited awareness about the need for psychotherapeutic intervention, geographically highly dispersed positions of the affected persons, unavailability of psychotherapeutic services or personnel in most places, and lack of resources. Clinical psychology professionals are not available in small towns and rural areas, so these therapeutic options are not available locally. To access these psychotherapy services survivors, have to travel long distances, taking long periods, which would have additional cost implications. In addition, most affected persons may not be able to afford the cost of psychotherapy or provide the time required for it considering their work commitments. Although the cultural aspects were not studied specifically, hesitancy in seeking and accepting intervention for psychological problems in the region has been reported, and one of the most common reasons is the stigma [4,12]. This might have contributed to service utilisation and participation in the study.

Facilitation of psychotherapeutic services

In the context of the above challenges, various approaches and methods can be adopted to meet the psychotherapeutic needs of the affected persons. It needs to be prioritized as rail and road accidents are fairly common in India [1], and most of the survivors are not receiving appropriate psychological support.

The survivors belonged to geographically highly dispersed areas and the unavailability of psychotherapy services or personnel can be dealt with by technologies involving online therapy, or telephone support. Many types of psychotherapies have been observed to be effective online and through the telephone. Remote assessment and management of common psychiatric disorders have been done effectively [13,14]. Although some of the survivors may need more intense and involved support and may have to attend a clinical setup in person, many therapies can be provided remotely. There are examples of remote support in India, e.g., the recent Tele-Mental Health Assistance and Nationally Actionable Plan through States (T-MANAS) programme [15], for mental health crisis management.

The lack of availability of psychotherapists requires training more personnel and expanding the services, however, that would be a long-term strategy. In the short term, this can probably be dealt with by enabling available personnel with training and task-shifting [16,17]. Some of the therapies can be provided by professionals other than psychologists, following training; this may include nurses, accredited social health activist (ASHA) workers, or other health care professionals in primary and secondary care setups. Many psychotherapeutic works can be done through these means and these are being practised elsewhere effectively [18]. This may be a short-term solution in resource-scarce regions.

One of the most important issues is that many survivors may not realize the stress symptoms and how that is affecting them. There is a need to inform and educate them about it along with informing them about the stress management strategies. As observed in this project psychoeducation was a key requirement for the survivors and their family caregivers. This information can be given during in-person sessions, over video, or telephone consultations. In addition, these can be shared through reading materials in the form of short leaflets posted to them.

The strategies in psychotherapy, that is, the types of psychotherapy methods to be used, need to be personalized according to the individual needs of the survivors. The strategies considered in this study are commonly used therapies in clinical scenarios for trauma survivors [11,19]. However, all these need to be individualized depending on the needs and associated conditions. Most of the therapies require multiple sessions and are provided in both individual and group sessions. These are used frequently in the clinical setups. However, considering the issues of unavailability of trained personnel and the challenges of survivors attending the clinical setups, there can be modifications of the type of therapies provided. Therapies can be manual-based, and culturally adapted to improve acceptably, along with reading materials in the local language as bibliotherapy. All these can be done under the supervision of psychologists through remote support using technologies [20]. There are many examples of the effectiveness of therapist support online or over the telephone [21,22]. This might reduce the number of sessions to be conducted in person by the psychologists. Brief interventions have also been suggested that may reduce distress [23]. Other solutions could be fewer sessions, single-session counselling, or therapies, which have been reported, however further research is required [24]. Examples of culturally valued relaxation methods are the breathing techniques in yoga and pranayama which can decrease the stress following a traumatic experience [25] and can be suggested for stress management [26], which most people in the region are aware of and practise.

In addition to trauma-related psychopathologies and traumatic symptoms, lots of other non-trauma factors needing psychological support were brought to focus by many survivors during their reviews. These included issues related to interpersonal relationships, marital problems, etc. These would need structured planning and additional specific therapies.

Along with the above, there is a need to support the family members who have been affected and report stress symptoms. Psychotherapeutic interventions should also be extended to other indirectly affected persons such as rescuers, and health professionals involved in the treatment of injured passengers; who experience psychological problems [12]. It has been reported that family, friends, and fellow passengers are extremely important during the recovery process, and such closeness should be promoted and facilitated [27]. Social support for the survivors and their families is also important for recovery from traumatic stress.

Strengths and limitations

This is the first study of this kind from India following a train accident, to our knowledge, exploring the psychotherapeutic needs of the accident survivors. It is expected that the results of the study will help address the knowledge gap in this area and arrange the largely unmet needs of the survivors. The qualitative, narrative approach with both cross-sectional and longitudinal components allowed a greater understanding of survivors' personal experiences and evolution of the psychological reactions to the trauma and helped in developing individualized psychotherapeutic strategies. The three-month post-incidence framework provided the advantage of a more elaborate perspective of the trauma and its medium-term impact on the survivors on various elements such as psychosocial, occupational, and interpersonal aspects. The case-based presentation provided a diversity of psychological responses to trauma. Assessments were carried out in the first language of the survivors, along with their family caregivers which facilitated and improved the validity of the assessments, and provided a broader picture of the impact of the trauma. These factors contributed further clarity on the specific therapeutic strategies required. The observations highlighted that instead of usual clinic-based mental health services, remote assessment and resource-appropriate interventions are strategies that may address disaster mental health needs in low-resource settings.

There are several limitations to this study. Compared to the number of survivors of the train accident, the sample size of this study is relatively small, which might affect the generalizability of the findings. Limiting factors of lower engagement were unavailability, inability to provide time for the sessions, and work-related concerns, besides the connectivity issues. Passengers who did not participate may be different from the study participants. The review sessions were conducted remotely over the phone, so the observation opportunity of face-to-face assessments and the component of ‘being there’ with someone emotionally distressed was not there. While it was helpful to have collateral history from family members in most cases, in a proportion of sessions there was a possible issue of not having privacy while interacting, which might have an influence. Standardized scales were not used to assess psychological distress or specific disorders, as the focus was to explore the concerns holistically to determine interventions. Similarly, particular psychiatric diagnoses of the participants were not arrived at during these psychological assessment sessions; these could have contributed to additional specific support. The study was conducted around three months after the accident, so the findings may not be generalizable to other timeframes, although trauma impacts may continue long term, the needs might be different.

## Conclusions

This exploratory narrative study looked at the psychotherapeutic needs of the 2023 Odisha train accident survivors. It was observed that there is a huge need for psychological support for the survivors and their family caregivers, and most of these are unmet. The types of psychotherapies needed are commonly available in the clinics; however, there are many challenges for survivors accessing those. Innovative strategies need to be explored to provide psychological support to the survivors. As the survivors are spread across a wide geographical region, and the availability of in-person psychological interventions is limited, tele-psychotherapy through telephone or online video consultations can be a viable option. In addition, local health professionals and community workers such as ASHA can be trained to provide initial psychological support which may be done locally and in person.

The psychological needs of the survivors should be managed effectively as most survivors would need long-term support, and without active in-reach, many survivors may not be followed up, which was observed in this study. Preferably the process of providing support should start early during the crisis period and continue as per the individual needs of the survivor. Techniques of intervention appear to be mostly supportive therapies, with elements of stress management, individualized relaxation methods, and psychoeducation regarding stress and its impacts, in the initial stages, followed by more trauma-focused therapies. Many survivors would require a range of therapies, depending on their individual needs; and it is expected that this support will be required for a long period. It is pertinent to establish a method to contact, engage, and support the survivors holistically; and ensure that psychological interventions should be an integral part of the support system as the needs are multiple and diverse with mental, physical, occupational, and interpersonal consequences after catastrophic trauma experience.

Future studies should look into the effectiveness of adapted methods of providing psychotherapy remotely, culturally modified therapies, and therapies provided by other colleagues trained for the purpose. As rail and road accidents are common, and there is an unmet need for psychotherapeutic support for a lot of survivors, a strategy addressing this may be developed by the health authorities and supported by the state.
